# Implant Treatment with 12-Year Follow-Up in a Patient with Severe Chronic Periodontitis: A Case Report and Literature Review

**DOI:** 10.1155/2019/3715159

**Published:** 2019-11-22

**Authors:** Keisuke Seki, Yoshiyuki Hagiwara

**Affiliations:** ^1^Implant Dentistry, Nihon University School of Dentistry Dental Hospital, Tokyo, Japan; ^2^Department of Comprehensive Dentistry and Clinical Education, Nihon University School of Dentistry, Tokyo, Japan

## Abstract

Tooth loss among adults is associated with progressive periodontitis. Implant prosthetic treatment has long been utilized in periodontal patients. Even when the implants are applied, ongoing management of periodontal disease and control of inflammation is necessary to maintain a healthy oral cavity. Lack of appropriate periodontal treatment can result in recurrence of periodontal disease during a maintenance period; loss of the supportive capacity of the periodontal tissues will increase the susceptibility of residual teeth to traumatic force. For this reason, it is worthwhile to improve oral function by applying implants as a fixed device. Here, we report that implant treatment in a patient with generalized severe chronic periodontitis helped maintain the periodontal and peri-implant tissue for a long term. We propose that initial periodontal treatment and ongoing supportive therapy can help maintain implants in patients with severe periodontitis. In addition, we reviewed case reports in the English literature so far.

## 1. Introduction

Periodontitis is an endogenous multibacterial infectious disease in which the periodontal tissues break down as a result of the interactions between specific anaerobic bacteria and host immune mechanisms [1–3]. Additionally, it is a multifactorial disease, involving bacterial, environmental, and biological factors [4]. For the periodontal treatment, it is effective for improving a local environment by removal of plaque retention factors while improving systemic factors like diabetes treatment and smoking cessation. Severe periodontitis is characterized by progressive destruction of the periodontal tissues resulting in over 5 mm of clinical attachment loss. A correlation between periodontitis and a weakened immune response has been reported, and the accumulated destruction of periodontal tissues with age can result in progressive attachment loss and bone resorption [5]. A recent study reported that severe periodontitis is the sixth most prevalent disease worldwide [6]. Due to the loss of teeth caused by this disease, the burden imposed on the physical, mental, and financial state of the patient increases. This also causes the patient's QOL to decrease markedly.

While a history of periodontitis is considered a localized risk indicator for implant failure at the start of maintenance, it has also been recognized as an important risk indicator for peri-implantitis [7]. It has been reported that a history of periodontitis decreases the success rate of implants. Therefore, appropriate periodontal treatment must be implemented for long-term implant stability [8]. In this regard, it is necessary to investigate previous reports related to long-term outcomes in severe chronic periodontitis patients with applied implant treatment. The purpose of this case report is to show the necessity of implant treatment for severe periodontitis and to include a review showing that the implant is useful for long-term conservation of residual teeth. The patient was maintained successfully for 12 years, suggesting that implants following initial periodontal treatment may contribute to maintaining good oral function.

## 2. Case Presentation

A nonsmoking 51-year-old woman visited Nihon University School of Dentistry Dental Hospital in April 2004 with a chief complaint of mobility in the maxillary teeth. The patient was in good general health. Her past medical and dental history was unremarkable, as was her family history. A few months before presentation, she developed difficulty eating as a result of tooth mobility and increased sensitivity to cold water.

### 2.1. Examination Findings

**Figure 1:**
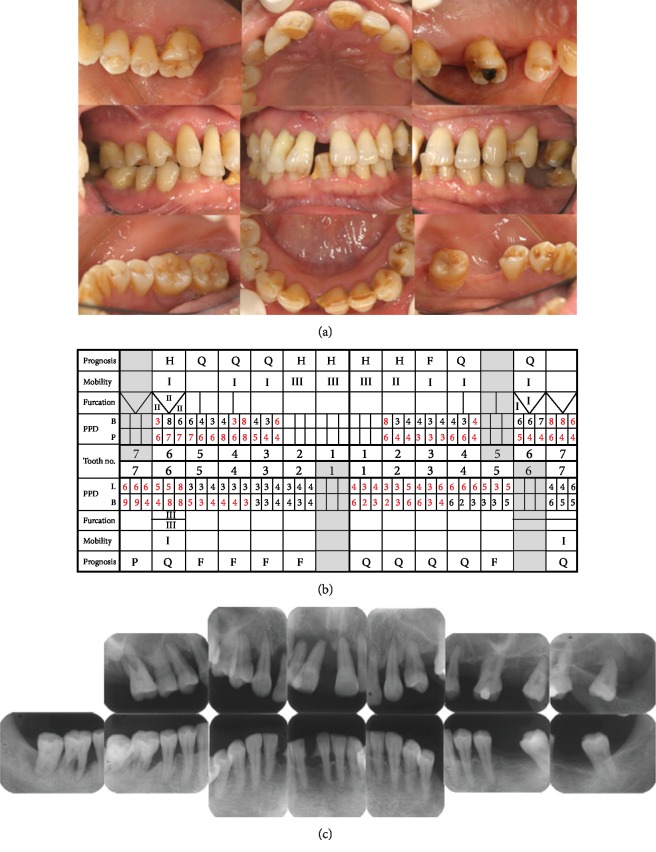
(a) Pretreatment intraoral photographs (April 2004). Generalized inflammation is observed, particularly between tooth #11 and tooth #12, where a fistula is present. (b) Pretreatment periodontal chart. Teeth #11, #12, #16, #21, and #22 were evaluated as “hopeless”; #46 and #47 were “poor” (PPD: periodontal probing depth; G: good; F: fair; Q: questionable; P: poor; H: hopeless, red: bleeding). (c) Pretreatment radiographs. Thirteen dental X-ray films show severe bone loss in the maxillary anterior and mandibular right molar regions.

On intraoral examination, diffuse redness and swelling were observed in the marginal gingiva and interdental papillae. Bleeding and purulent discharge from the pockets were found mainly in the right molar region. The patient had halitosis. The deposition of supragingival calculus was observed in the mandibular anterior segment. Pathologic tooth migration was observed in teeth #11 and #12 (overjet 5 mm, overbite 7 mm) (Figure 1(a)). Teeth #11, #12, and #21 exhibited class III mobility. Tooth #46 had a class III furcation lesion. Sixty-one percent of the teeth had a deep periodontal probing depth (PPD) over 4 mm (PPD ≥ 4), 61% had bleeding on probing (BOP) (Figure 1(b)), and all teeth scored 100% on O'Leary's plaque control record. Radiographic examination revealed periodontal tissue destruction, generally with 3 to 5 mm of horizontal bone resorption and 4 to 6 mm of vertical bone resorption in teeth #16, #14, #26, and #46 (Figure 1(c)). Occlusal examination showed Eichner's index B1. Based on these results, we diagnosed generalized moderate-to-severe chronic periodontitis.

### 2.2. Treatment Plan

The treatment plan consisted of the following items:

1. Initial periodontal treatment
  - Oral hygiene instruction
  - Scaling and root planing
  - Tooth extraction (teeth #16, #12, #11, #21, #22, and #27)
  - Therapeutic partial denture (teeth #12–#22)
  - Root canal treatment and hemisection (tooth #46)
  - Occlusal adjustment (teeth #13 and #23)
2. Reevaluation
3. Treatment of oral functional rehabilitation
  - Implant prostheses (site of #16, #12, #22, #36, and #41)
4. Reevaluation
5. Supportive periodontal and implant therapy

### 2.3. From the First Visit to Initial Periodontal Treatment (2004–2005)

**Figure 2:**
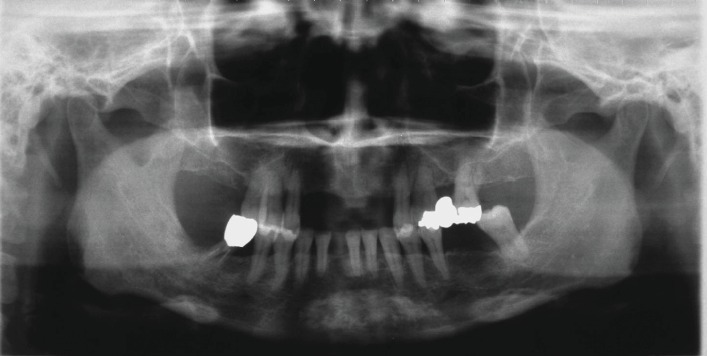
Reevaluation radiographs. Panoramic radiography revealed partially edentulous maxilla and mandible.

Prior to initial periodontal treatment, we informed the patient of her current periodontal status and the necessity of treatment. The patient gave her consent to the treatment plan. We instructed the patient to use the Bass brushing method and recommended using dental floss or a tufted toothbrush. Following gross scaling, confirming that redness and swelling had improved, the teeth of chief complaint (teeth #11, #12, and #21) were extracted. A therapeutic partial denture was prepared and applied immediately. Scaling and root planing were performed under local anesthesia for all teeth with PPD over 4 mm. At the first reevaluation, the patient's oral hygiene had improved (BOP: 12%; PPD ≥ 4: 12%; PCR: around 20%). However, tooth #47 was determined to be unsalvageable and was extracted. Root canal treatment was performed for the distal root of tooth #46; while the medial root was prognosed to be impossible to preserve due to furcation involvement, trisection was adopted (Figure 2).

### 2.4. Implant Treatment (2005–2007)

Implants were chosen respecting the patient's desire to avoid using a removable device and further cutting of natural teeth. We created a diagnostic template based on setup models and used it at X-ray and CT examination. Following administration of local anesthetic (Xylocaine®; 2% lidocaine with 1 : 100,000 epinephrine, DENTSPLY SANKIN, Tokyo, Japan), an incision was made on the alveolar crest and a full-thickness flap was raised. Root form-type implants (Replace® Select Tapered; Nobel Biocare AB, Göteborg, Sweden) were placed with the aid of surgical guides (at #41: NP 3.5 × 13 mm; at #36: WP 5.0 × 10 mm; at #12 and #22: RP 4.3 × 13 mm). Considering the lack of maxillary bone at the site of #16, a 4.0 × 8 mm implant (4.0ST; ASTRA TECH AB, Mölndal, Sweden) was installed using osteotome sinus floor elevation. Primary stability was achieved for all implants. The sites were closed with interrupted 5-0 sutures (MONOCRYL® 5.0; Ethicon, Inc., Somerville, NJ, USA). After these procedures, medications (cephalexin, 1000 mg twice daily for 3 days and diclofenac, 50 mg, three times daily as needed for pain) and postoperative care were prescribed. No adverse postoperative sequelae were observed.

All surgical procedures were conducted with a two-stage surgical approach. During the 5-month relief period, a partial denture was used in the maxillary anterior region. With regard to the reevaluation for 3 months later in the mandibular, abnormal findings such as drainage or bleeding were not observed.

**Figure 3:**
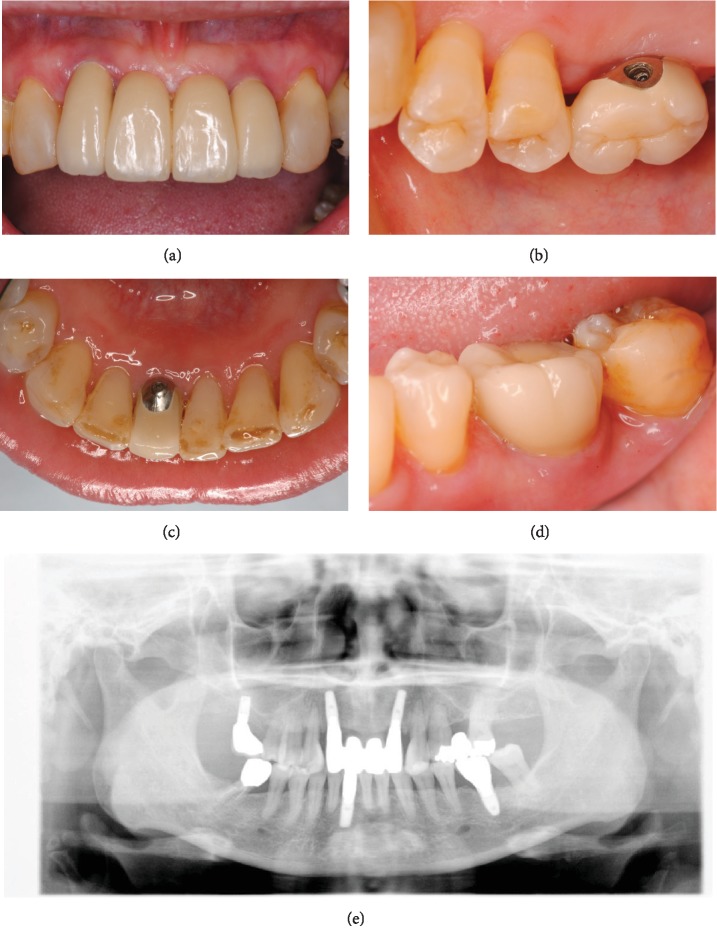
Clinical situation at the delivery of prosthetic rehabilitation: (a) at #12 and #22, (b) at #16, (c) at #41, (d) at #36, and (e) postoperative radiographs.

**Figure 4:**
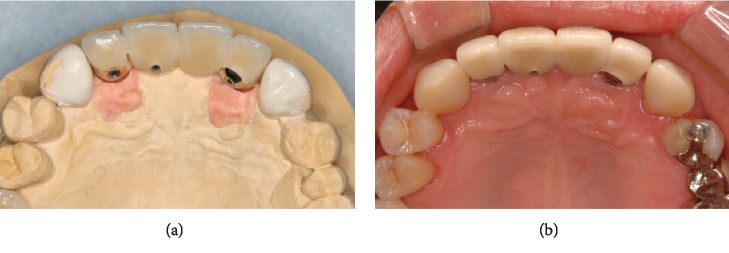
Composite resin was built up on palatal aspect of both canines: (a) setup model and (b) posttreatment.

Secondary surgery was performed after an adequate healing period, with confirmed osseointegration during surgery. After 1 month of soft tissue healing, the superstructure was made. All custom abutments and porcelain-fused-to-metal crowns were retained with side screws (Figures 3(a)–3(e)). In addition, the palatal surface at sites #13 and #23 was adjusted, molding both in composite resin, to obtain disclusion during lateral movement, according to the pretreatment setup model (Figure 4).

### 2.5. Supportive Periodontal and Implant Treatment (2007–2018)

After superstructure setting, the patient was given supportive periodontal and implant therapy and scheduled for follow-up appointments at 3-month intervals which is the most appropriate time to destroy the microflora in the periodontal pocket. Throughout the follow-up period, probing depths of both natural teeth and implants were monitored carefully. For the areas of plaque accumulation, we instructed the patient on the use of interdental brushes (Interprox® Plus; DENTAID, Barcelona, Spain) and enhanced her oral hygiene with regular professional cleanings. To remove dental plaque, we used hand instruments for the implants and an ultrasonic scaler for natural teeth. For professional tooth cleaning, a polishing paste not containing fluoride (Hawe Implant paste®; KerrHawe SA, Bioggio, Switzerland) was used in regard for avoiding titanium corrosion. Full-mouth X-rays were taken every 2 years, and changes in the peri-implant tissue were monitored. In natural teeth, we monitored the development of new bone resorption and root caries.

**Figure 5:**
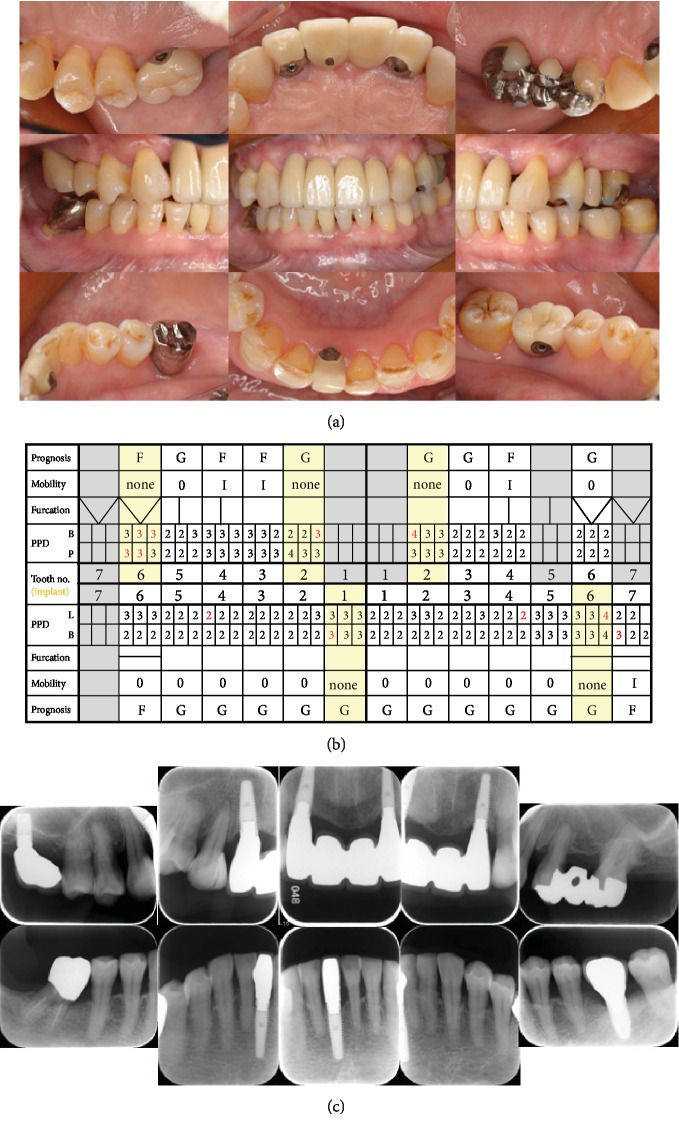
(a) 12-year postoperative intraoral photographs (April 2016). Intraoral view after completion of care. The gingival texture and peri-implant mucosa appear healthy. (b) 12-year postoperative periodontal chart. Prognoses ranged from “fair” to “good” (PPD: periodontal probing depth; yellow: implant). (c) 12-year postoperative radiographs. Posttreatment radiographs suggest improvement of the severe bone resorption lesions. No bone resorption is seen around the five implants.

We did not observe inflammatory signs such as redness, swelling, pus, or other dysfunctions in the peri-implant mucosa during the maintenance period (Figure 5(a)). Periodontal pockets have been maintained with less than 3 mm PPD and below 10% BOP in natural teeth (Figure 5(b)). All implant sites have maintained PPD of 3 to 4 mm, with slight BOP observed. The modified Silness–Löe plaque index was below a score of 1 for all implants. No pathological finding of remodeling surrounding implants was recognized on radiographic examination (Figure 5(c)). In oral microbiological examination using quantitative real-time PCR, *Porphyromonas gingivalis* was below the detection limit. With regard to prosthetic complications, the patient experienced tipping of the porcelain at site #31 shortly after starting function; because this tipping did not cause problems, we followed the course. Similarly, the abutment screw at site #16 loosened twice during the 12-year maintenance period. In each case, the screw was restored after cleaning and occlusal adjustment. The patient's oral hygiene has been well maintained, with no recurrence or worsening of periodontal disease among natural teeth.

## 3. Literature Review

The present study searched for articles published until 2018. The database Medline via PubMed was searched using the following terms: (“Dental Implants” [Mesh] OR “Dental Implantation” [Mesh]) OR (“Dental Prosthesis, Implant-Supported” [Mesh] OR implant [Title/Abstract]) AND (periodontitis [MeSH Terms] OR periodontitis [Title/Abstract]) AND (case reports [Publication Type] OR “case reports” [Title/Abstract] OR “case report” [Title/Abstract]) AND long-term [Title/Abstract]. Case reports on rare diseases or specific syndrome and edentulous patients and literature except for English were omitted with manual search. Six case reports were hit in the past 12 years (Table 1). Many reports showed that the majority of the interval maintenance period was 3 months [9–16]. In these reports, obtaining favorable healing process following periodontal initial treatment and surgical procedure was mentioned.

## 4. Discussion

This report describes the successful implant treatment and long-term management of a patient with severe periodontitis. To achieve successful periodontal treatment, both the removal of inflammatory factors and the improvement of occlusal factors are needed. In this case, these goals were obtained through oral hygiene instruction, scaling and root planing, occlusal adjustment, and application of a partial denture. A supportive therapy after active periodontal therapy is effective in maintaining the health of periodontal tissues [17].

Comprehensive treatment including maintenance or supportive therapy contributes to a decreased incidence of tooth loss [18]. A previous study found that the incidence of tooth loss among periodontal patients ranged from 0.09 to 0.16 per year [19–21]. That finding suggests that tooth loss can be minimized to only one tooth every 10 years with maintenance treatment. In this case, six hopeless teeth were extracted out of 24 present at the initial visit. Five implants were placed as prosthesis for seven missing teeth, and the patient did not lose any teeth during the maintenance period. In this regard, our results were better than those of similar reports.

To improve Eichner's index from B1 to A1, the occlusal supporting area was increased while stability of the periodontal tissue was achieved. Due to improved Eichner's index from B1 to A1, the occlusal supporting area was increased while stability of the periodontal tissue was achieved. It was prospected that periodontal tissue was protected by applying implants to provide a vertical stop and proper anterior guidance. In contrast, the reason for the loosening of the abutment screw was considered to be the wearing of the composite resin, which altered the occlusal condition and lateral forces on the superstructure at site #16.

It has been reported that long-term maintenance therapy for implants can prevent other complications and improve success rates [22]. Among the frequent problems with implants, prosthetic and biological complications are common [23]. In particular, peri-implantitis has gained attention as one major biological complication in recent years. *Aggregatibacter actinomycetemcomitans*, *Porphyromonas gingivalis*, *Prevotella intermedia*, and *Fusobacterium* spp. are implied to be pathological bacteria related to peri-implantitis. Likewise, their role in periodontitis has been studied [24, 25]. Moreover, natural teeth can act as reservoirs of microorganisms for colonization of oral implants [26].

A reliable treatment of peri-implantitis has not yet been established, and managing peri-implant tissue remains difficult. Nevertheless, it is clear that the risk factors for peri-implantitis include poor oral hygiene, a history of periodontitis, and smoking [7]. While the mechanisms and pathology of peri-implantitis are similar to those of periodontitis [27], much remains unknown about the condition [28]. Although periodontitis has been reported to decrease implant success rate [29], peri-implantitis can be prevented among patients with severe periodontitis. This success mainly relies on appropriate initial periodontal treatment, which allows us to maintain the patient's oral health during long-term maintenance therapy [30].

In this case, inflammatory remarkable symptoms were not detected during the maintenance period. Although it was previously established that BOP is evidence of inflammation [31], implant BOP is an uncertain clinical parameter for diagnosing the health of these tissues because of greater sensitivity than natural teeth [32] and depends on the shape of the superstructure and measuring technique. However, although it is not a completely established method, implant probing and accompanying bleeding are considered useful for diagnosing peri-implantitis [33]. Moreover, the absence of periodontal bacteria during supportive therapy confirmed that removal of inflammatory factors was successful and continuous.

In the present case, we eliminated inflammatory factors and improved the oral environment with comprehensive periodontal treatment. In the future, careful monitoring of alteration of occlusion and trauma force for teeth with altered crown-root ratios will be necessary. By applying implants to stabilize occlusion, the mobility of natural teeth was decreased. The course of this patient is good, with no deep PPD. Ongoing supportive therapy is necessary.

## 5. Conclusion

The patient diagnosed with generalized severe chronic periodontitis underwent comprehensive treatment involving implants. A long-term maintenance has been achieved, with no recurrence of periodontitis and with stability of the peri-implant tissues. With appropriate initial periodontal treatment and ongoing supportive therapy, oral health can be maintained soundly and longitudinally in patients with implants.

## Figures and Tables

**Table 1:** Case reports of implant treatment in a patient with periodontitis.

Authors (y)	Patient	Diagnosis	Therapy	Implants (*n*)	Maintenance interval	Follow-up (y)
Brezavšček et al. (2014)	71 M	Chronic generalized periodontitis (severe form)	(i) Extraction of 16 hopeless teeth (ii) Scaling and root planing (iii) Temporary RPDs (iv) Tooth- and implant-supported telescope-retained PRDs	Astra® (2)	3 months	1

Zafiropoulos et al. (2011)	39 F	Severe periodontitis	(i) Initial treatment, periodontal surgery (previous dentist) (ii) Extraction of hopeless tooth	Straumann® (12)	3-6 months	7

Zafiropoulos and Rebbe (2010)	47 M	Advanced chronic periodontitis	(i) Scaling and root planing (ii) Antibiotic treatment (iii) GTR (iv) Extraction of 7 teeth after 2 years (v) Overdenture, PFM crown (vi) 1 tooth fracture during 9-year maintenance	Straumann® (6)	6 months	15

Naert and Quirynen (2007)	47 F	Diagnosed “full extraction”	(i) Comprehensive treatment (Department of Periodontology, 30 years before) (ii) Recurrence of tooth fracture	NR (5)	NR	28

Kreissl (2007)	51 F	Severe periodontitis	(i) Extraction of hopeless tooth (ii) CTG	XIVE (5)	NR	NR

Hofer et al. (2002)	22 M	Rapid generalized early-onset periodontitis (1993 AAP definition)	(i) Scaling, gingivectomy (previous dentist) (ii) Hygiene phase, FOP (referred specialist)	Straumann® (4)	3 months	2

RPD: removable partial denture; GTR: guided tissue regeneration; PFM: porcelain-fused-to-metal; CTG: connective tissue graft; FOP: flap operation; NR: not reported.
